# Responses of Arsenic and Soil Properties to Remediation: Evidence from a Two-Year Monitoring Study in an Abandoned Gold Mining Area

**DOI:** 10.3390/toxics14040316

**Published:** 2026-04-08

**Authors:** Zengling Tang, Lingyun Li, Yingyuting Li, Huayi Chen, Yili Zhang, Tian Hu, Zheng Hu

**Affiliations:** 1 Guangdong Research Center for Agricultural Soil Pollution Prevention and Control Engineering Technology, College of Natural Resources and Environment, South China Agricultural University, Guangzhou 510642, China; 2 School of Tropical Agriculture and Forestry, Hainan University, Haikou 570228, China; huayi93@hainanu.edu.cn; 3 School of Environmental Science and Engineering, Hainan University, Haikou 570228, China

**Keywords:** mining soil remediation, As mobility, soil physicochemical properties, temporal dynamics

## Abstract

Arsenic (As)-enriched soils in abandoned mining areas pose persistent environmental risks, yet the temporal evolution of remediation is rarely evaluated. In this study, a two-year field monitoring program was conducted at a severely As-contaminated abandoned gold mine in Guangdong Province, China, to examine the temporal dynamics of soil properties and As behavior under different remediation strategies. Three representative slopes were investigated: slope A (slope reshaping and revegetation), slope B (terraced engineering interception), and slope C (an area influenced by acidic water bodies). The results showed that both total and available As at slopes A and B exhibited a similar pattern of initial increase followed by decline and stabilization, indicating a clear temporal scale for remediation effects. Slope A exhibited greater spatial variability, whereas slope B showed relatively minor fluctuations, suggesting that terraced engineering measures contributed to enhanced As stability. In contrast, slope C had lower total As but a higher proportion of available As prior to remediation due to the acidic conditions. Following remediation, both total and available As at slope C decreased markedly and remained stable for about six months; however, a rebound trend was observed after approximately 1.5 years, indicating the time-limited effectiveness of passivation treatments. Specifically, total As at slope C decreased from 22,916 to 4011 mg·kg^−1^, accompanied by a 65–85% reduction in available As. Meanwhile, soil pH, soil organic matter, and cation exchange capacity exhibited pronounced non-linear variations, with an overall tendency to recover toward pre-remediation conditions. These findings underscore the importance of long-term monitoring for evaluating remediation effectiveness and periodic assessments (e.g., semiannual monitoring of soil As and nutrient status) to support adaptive environmental management and optimization of remediation strategies.

## 1. Introduction

The long-term exploitation of mineral resources has supported regional economic development, yet it has also imposed persistent pressure on local ecosystems [1,2]. Large volumes of waste rock, tailings, and contaminated wastewater are commonly generated during mining and beneficiation processes. These materials are often deposited on land surfaces for extended periods, gradually reshaping mining areas into ecosystems dominated by solid waste [3,4,5]. With prolonged exposure to weathering, rainfall, and hydrological processes, soil systems in these areas undergo continuous disturbance. As a result, soil structure becomes degraded, nutrient balance is disrupted, and ecosystem functions progressively decline. In many abandoned or poorly managed mining sites, these changes accumulate over time, leading to fragile ecological conditions characterized by long-term instability and limited resilience [5,6,7].

Heavy metal contamination is one of the most critical environmental challenges in mining areas. Mine wastes are often enriched with toxic elements such as cadmium (Cd), arsenic (As) and lead (Pb), which can be gradually released from solid phases through weathering, rainfall infiltration, and hydrological transport [7,8,9,10]. Once mobilized, these metals migrate into surrounding soils via runoff or percolation, posing long-term environmental risks. The transport and transformation of these heavy metals (lloids) are controlled by multiple factors, including soil physicochemical properties, rainfall intensity, and topography [11,12,13]. Among these contaminants, As is of particular concern due to its widespread occurrence in metal mining areas and its strong sensitivity to soil pH, redox conditions, and the presence of iron and manganese oxides [8,14,15,16,17]. Such widespread occurrence largely stems from the association of As with sulfide minerals in ore bodies (e.g., FeAsS, Cu_12_As_4_S_13_) [18,19]. Mining activities expose these As-bearing phases to atmospheric and hydrological conditions, enhancing oxidative weathering and facilitating the release of arsenic into surrounding environmental media. Changes in soil environmental conditions following remediation may induce As reactivation, increasing its mobility and bioavailability and posing potential risks to agroecosystem stability and food safety.

Current remediation practices in mining areas typically combine engineering measures, soil amendments, and ecological restoration to reduce metal mobility and improve soil quality [20,21,22]. Such approaches can effectively lower metal (lloid) bioavailability in the short term by modifying soil physicochemical properties, restricting contaminant migration, and enhancing sorption capacity. However, increasing evidence suggests that the effectiveness of remediation is not static [23]. The long-term stability of these approaches is strongly influenced by subsequent changes in soil structure, hydrological regimes, and the aging or transformation of amendment materials. For example, routine monitoring of the Brumadinho tailings dam in Brazil focused primarily on surface deformation, while pore-water chemistry and redox dynamics remained largely unexamined. Progressive sulfide oxidation within the tailings led to acidification and a decline in shear strength, and ultimately catastrophic failure killed 270 people in January 2019 [24,25]. In another case, at the Carnoulès high-arsenic acid mine drainage site in France, seasonal wet–dry cycles raised the exchangeable As(V) fraction of freshly formed amorphous Fe-As precipitates from 20% to 45% within six months [26,27]. Despite these insights, most studies still emphasize immediate remediation outcomes, whereas the post-remediation temporal dynamics of contaminant behavior and potential risk, particularly the fluctuations that may occur over a period following engineering intervention under heterogeneous geomorphological and hydrological conditions, remain insufficiently documented. Consequently, continuous field-based evidence on post-remediation arsenic dynamics is still limited.

Therefore, this study specifically focuses on As as the target contaminant and a two-year field monitoring program was conducted at an abandoned gold mining site in Guangdong Province, China, characterized by As-enriched soils, with the aim of systematically evaluating the temporal evolution of soil environmental quality before and after remediation. Within this site, three representative slope units with contrasting geomorphological and environmental characteristics were selected, including a gently sloping area with relatively low disturbance (A), a heavily disturbed stepped slope (B), and an acid-affected slope influenced by surface runoff (C). Changes in soil As concentrations and associated physicochemical properties were systematically monitored to reveal the temporal dynamics of soil evolution following remediation. The results of this study provide scientific support for evaluating the effectiveness and long-term stability of mine reclamation measures and also offer important insights into the potential risks posed to surrounding agricultural soils as well as the source identification and migration behavior of heavy metals in mining-affected areas.

## 2. Materials and Methods

### 2.1. Study Area

**Figure 1:**
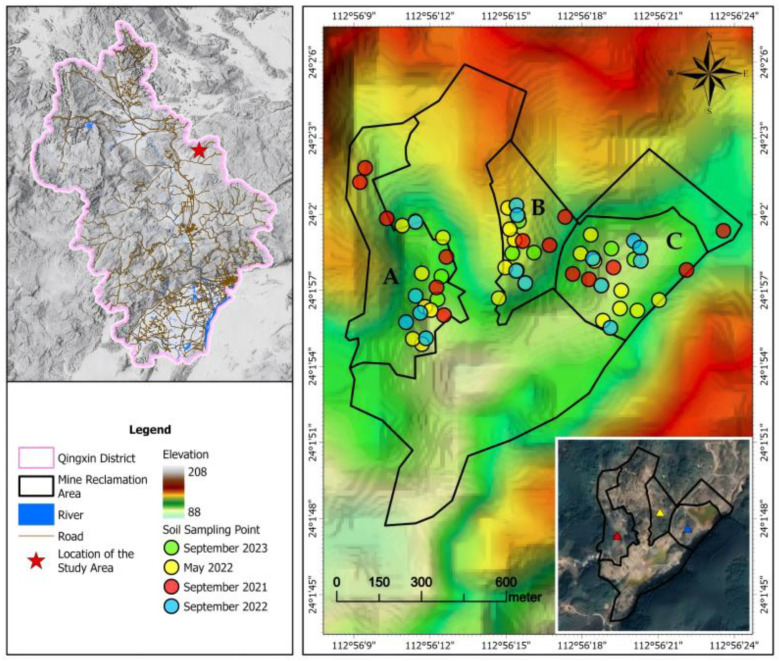
Topographic contour map of the study area. A, B, and C represent three different slopes; different colored circles indicate sampling time points as follows: yellow for September 2023, green for May 2022, red for September 2021, and blue for September 2022.

The study area is located at a reclaimed gold mine tailings site in Guangdong Province, China (Figure 1). The study area is characterized by a typical subtropical monsoon climate with warm and humid conditions, an average annual temperature of approximately 20–22 °C, and a frost-free period exceeding 330 days. Precipitation is abundant, with a multi-year average of approximately 2139 mm, but shows pronounced seasonal variability, with about 80% of annual rainfall occurring during the wet season (April–September), often in the form of intense rainfall events. The mean annual evaporation ranges from approximately 1200 to 1300 mm, and annual precipitation generally exceeds evaporation, with roughly 70% of rainfall contributing to runoff.

This mining area covers approximately 2.19 × 10^5^ m^2^ with a maximum elevation of about 206 m, where gold is the primary resource accompanied by associated minerals such as rare earth elements, tin, lead, and zinc. In addition to native and oxidized gold, part of the gold occurs in sulfide-associated forms hosted in minerals such as pyrite (FeS_2_) and arsenopyrite (FeAsS). Historical unregulated mining and primitive gold extraction activities in the area left behind large amounts of waste residues rich in arsenic and sulfur, which constitute an important source of the severe As contamination observed at the site. The local government took a series of measures in 2020 to mitigate environmental risks and restore the ecological functions of the abandoned mining area.

The region is characterized by hilly terrain with pronounced topographic variation, and the surrounding land is mainly used for agricultural production, with rice as the dominant crop and a small proportion of upland crops such as sweet potato. Three slope areas (A, B, and C) were selected to represent the major geomorphological, contamination, and hydrological gradients within the mining site. These units typify low-relief amended areas (A), heavily disturbed terraced areas with tailings (B), and hydrologically influenced acidic zones (C), thereby capturing the spatial heterogeneity of the site for comparative monitoring. The detailed characteristics and remediation measures for each area prior to restoration are summarized in Table 1 and Table S1.

### 2.2. Sample Collection and Analysis

Soil sampling was conducted at four stages of the remediation process (T1–T4), corresponding to one year before remediation (T1), immediately after remediation (T2), six months after remediation (T3), and one and a half year after remediation (T4), with sampling dates of 11 September 2021, 5 May 2022, 13 September 2022, and 27 September 2023, respectively. At each sampling stage, soil samples were simultaneously collected from slopes A, B, and C, which were selected to reflect the heterogeneous environmental conditions and remediation responses of the mining area. The collected soil samples were analyzed for As concentrations and key physicochemical properties to evaluate temporal changes in mine soil before and after remediation.

#### 2.2.1. Sample Collection

Soil sampling was conducted in accordance with the protocols specified in NY/T 395-2012 [28]. Within each slope area, three sampling points were distributed in a serpentine pattern to ensure representative sampling. At each sampling point, surface soil samples were collected from a depth of 0–20 cm using a five-point composite sampling method. The subsamples from each point were thoroughly mixed to form a composite sample with a total mass of no less than 1 kg. After collection, soil samples were placed in polyethylene self-sealing bags and transported to the laboratory. Plant residues, stones, and other debris were manually removed, and the samples were air-dried at room temperature. The dried samples were ground and passed through 2 mm, 1 mm, and 0.149 mm nylon sieves for subsequent analyses. All soil samples were analyzed in triplicate.

#### 2.2.2. Analysis of Soil Physicochemical Properties

Soil physicochemical properties were determined following standard soil agrochemical procedures. Soil pH was measured using a glass electrode method at a soil-to-water ratio of 1:2.5 (*w*/*v*) with a pH meter (PHS-2F, Leici, Shanghai, China) in accordance with NY/T 1377-2007 [29]. Soil organic matter (SOM) was determined using an elemental analyzer (Vario MICRO cube, Elementar Analysensysteme GmbH, Langenselbold, Germany) following the Chinese agricultural standard NY/T 1121.6-2006 [30]. Cation exchange capacity (CEC) was determined by the ammonium acetate method according to LY/T 1243-1999 [31]. Alkali-hydrolyzable nitrogen (AN) was determined by the alkaline hydrolysis diffusion method (LY/T 1228-2015) [32]. Available phosphorus (AP) and available potassium (AK) were measured using a UV–Vis spectrophotometer (UV-2600, Shimadzu, Kyoto, Japan) and an atomic absorption spectrophotometer (novAA350, Analytik Jena, Jena, Germany), respectively, according to NY/T 1121.7-2014 and NY/T 889-2004 [33,34]. Detailed analytical procedures are provided in the Supplementary Materials.

#### 2.2.3. Determination of Soil As Content

Total As in soil was determined by mixed-acid digestion (HF–HCl–HNO_3_–HClO_4_) followed by atomic fluorescence spectrometry (AFS, AFS-933, Beijing Jitian, Beijing, China), according to GB/T 22105.2-2008 [35]. A 50 mL polytetrafluoroethylene (PTFE) tube was immersed in an acid bath (10% nitric acid) for at least 24 h, followed by rinsing with ultrapure water and drying. Subsequently, 0.1000–0.3000 g (accurately weighed to 0.001 g) of soil was weighed into the PTFE tube using a ten-thousandth analytical balance. Then, 6 mL of concentrated nitric acid, 3 mL of concentrated hydrochloric acid, and 2 mL of hydrofluoric acid were sequentially added. The tube was tightly sealed and left overnight in a fume hood. It was subsequently fixed in a microwave digestion system, and the experimental temperature was programmed as follows: first, heating to 120 °C with the lid on for 1 h; then removing the lid until the evolution of brown fumes ceased; followed by adding perchloric acid and raising the temperature to 175 °C until the solution turned pale or orange-yellow; and finally, adding 1 mL of nitric acid to continue digestion until the solution became colorless or light yellow. After the digestion system cooled to room temperature, the PTFE tube was rinsed repeatedly with small volumes of ultrapure water, and the solution was transferred and diluted to a final volume of 25 mL in a volumetric flask. After standing for 0.5 h, the solution was filtered into a 10 mL centrifuge tube for further analysis.

Available As was extracted using 0.05 mol·L^−1^ NH_4_H_2_PO_4_ solution [36,37]. Briefly, 1.00 g of air-dried soil passed through a 2 mm sieve was placed into a 50 mL polyethylene bottle, followed by the addition of 25 mL of NH_4_H_2_PO_4_ extractant. The mixture was shaken at 250 rpm for 16 h at 25 °C and then filtered and diluted prior to analysis. The As concentration in the extract was determined using AFS under the same conditions.

The sequential extraction of arsenic in soil was performed according to a five-step procedure: Step 1 involved extraction of the non-specifically adsorbed fraction using ammonium sulfate solution (0.05 mol/L); Step 2 extracted the specifically adsorbed fraction with ammonium dihydrogen phosphate solution (0.05 mol/L); Step 3 targeted the amorphous iron oxide-bound fraction using ammonium oxalate solution (0.2 mol/L); Step 4 involved extraction of the crystalline iron oxide-bound fraction using a mixture of ammonium oxalate (0.2 mol/L) and ascorbic acid (0.1 mol/L); and Step 5 determined the residual fraction by digesting the solid residue with a mixture of hydrochloric acid, nitric acid, hydrofluoric acid, and perchloric acid.

### 2.3. Data Analysis

All experimental data were processed and analyzed using Microsoft Excel 2019 and IBM SPSS Statistics 26. Results are expressed as mean ± standard deviation (SD). Statistical differences among treatments were evaluated using Duncan’s multiple range test at a significance level of *p* < 0.05. Graphs were generated using Origin 2021. Map visualization was performed using ArcGIS Pro 3.1.6. All measurements were performed in triplicate to ensure data reliability.

## 3. Results and Discussion

### 3.1. Changes in Soil As Content

**Figure 2:**
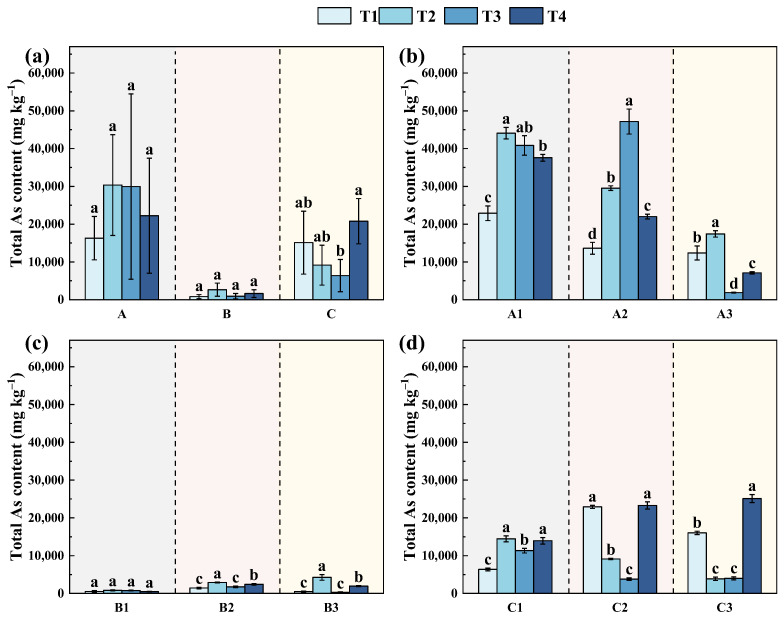
Total As content at different sampling stages: (**a**) overall comparison among slopes A–C; (**b**) slope A; (**c**) slope B; and (**d**) slope C. T1, one year before remediation; T2, immediately after remediation; T3, six months after remediation; T4, one and a half years after remediation. A1–A3, B1–B3, and C1–C3 denote the three sampling points on slopes A, B, and C, respectively. Different lowercase letters indicate significant differences among sampling times at the same site (*p* < 0.05).

The monitoring results revealed a clear spatial difference in total As concentrations across the study area. As levels were highest in slope A, ranging from 16,288–29,952 mg·kg^−1^, followed by slope C (6383–20,780 mg·kg^−1^), while slope B showed markedly lower values (810–2657 mg·kg^−1^) (Figure 2a). This uneven distribution is the combined influence of site contamination legacies and different remediation strategies across the study area. Slope A is characterized by relatively gentle terrain and limited elevation variation, where a thick layer (~10 cm) of bluish-black contaminated residues remained before remediation (Table 1). These conditions favored As accumulation in surface soils. Although soil removal and stabilization were adopted, As levels remained elevated. This pattern is mainly attributed to the disturbance of As-bearing subsurface layers during remediation, which promoted the upward redistribution of As into the surface soil [14,26]. In contrast, slope C is located adjacent to an acidic water body and strongly influenced by hydrological processes. Under acidic conditions, As is more readily released from mineral phases, while hydrological processes such as runoff and water exchange impose opposing controls through dilution and localized accumulation, giving rise to moderate As levels [38,39,40]. Slope B consists of terraced slopes that underwent systematic engineering remediation, exhibiting the lowest total As level among the three slopes. Large-scale reshaping of the slope surface altered the original soil structure and material distribution [20,40,41]. These changes promoted the redistribution of contaminants and reduced their retention in surface soils, thereby limiting As accumulation.

Temporal variations in total As at each slope were evaluated to further clarify these differences (Figure 2b and Figure S1a). A comparison between the initial sampling stage (T1) and the final stage after 1.5 years of remediation (T4) showed that total As rebounded to levels exceeding those prior to remediation at certain locations, with the maximum increase reaching 14,690 mg·kg^−1^ (Figure S1a). Such accumulation is likely associated with the geomorphological characteristics of areas with relatively small elevation gradients, where runoff-driven transport from upslope regions can facilitate the lateral migration and deposition of metal-bearing materials [42,43,44]. Moreover, pronounced spatial variability in As concentrations was observed among the three sampling sites on slope A. Prior to remediation, the slope surface was covered by an approximately 10 cm thick layer of bluish-black contaminated residues, whose distribution was likely heterogeneous due to historical deposition and subsequent disturbance. In addition, remediation activities were implemented with spatially variable intensity (e.g., differences in excavation, reshaping, and material redistribution), which may have further amplified local differences in contamination levels across the slope [40,42,43,44]. Total As levels at all sites increased markedly immediately after remediation (T2), indicating a pronounced short-term disturbance and redistribution effect associated with soil excavation, mixing, and material relocation during remediation, as well as possible exposure of previously buried As-rich materials. However, the subsequent evolution differed among sampling points. As levels gradually declined after T2 at A1, suggesting progressive stabilization following the initial disturbance. In contrast, A2 exhibited a delayed peak, with As continuing to increase at T3 before declining at T4, reflecting a stronger influence of subsurface material exposure and slower post-remediation equilibration. A3 showed a relatively moderate increase at T2 followed by a sharp decrease at T3 and partial recovery at T4, indicating higher sensitivity to local hydrological or surface disturbance conditions. These results demonstrate that although remediation initially induced As enrichment across all sites, the subsequent changes in As levels differed markedly.

Between T1 and T4, total As at slope B changed modestly (−26 to 1473 mg·kg^−1^) and was lower than that at slopes A and C throughout the monitoring period (Figure S1b and Figure 2c). Despite this pattern, As levels at slope B exhibited a clear but non-monotonic response to remediation. Prior to remediation (T1), As levels were relatively low. Following engineering intervention (T2), concentrations increased markedly, with all sampling points showing enrichment of approximately 1.6–8.7 times compared with that at T1. This increase was contributed to the effects of slope reshaping and soil reworking, which likely promoted the exposure and redistribution of As-bearing materials from deeper layers to the surface. Subsequently, As levels declined at T3 as slope structures gradually stabilized and disturbance-induced material transport weakened. At T4, a moderate rebound was observed at some sites; however, As levels remained lower than those measured immediately after remediation.

At slope C, total As increased at all sampling sites 1.5 years after remediation (T4), with increments ranging from 377 to 7581 mg·kg^−1^ (Figure 2d and Figure S1c). Following remediation, total As declined substantially during the early stage (T2–T3), particularly at C2 and C3, where concentrations decreased from 22,916 and 16,033 mg·kg^−1^ to below 3807 and 4011 mg·kg^−1^, respectively, representing reductions exceeding 70%. This pronounced decrease is likely attributable to the combined effects of alkaline amendment application and hydrological flushing, which increased soil pH and facilitated the mobilization and removal of part of the As-bearing materials [45,46]. However, this decreasing trend was not sustained over time. At the one-year stage (T4), total As increased again at all three sample sites, with concentrations rising to 13,946, 23,294, and 25,101 mg·kg^−1^ at C1, C2, and C3, respectively. This phenomenon is mainly associated with the strong hydrological influence in slope C, where prolonged rainfall, fluctuating water tables, and acidic conditions facilitate As re-mobilization from underlying sediments or adjacent contaminated zones [17,42].

**Figure 3:**
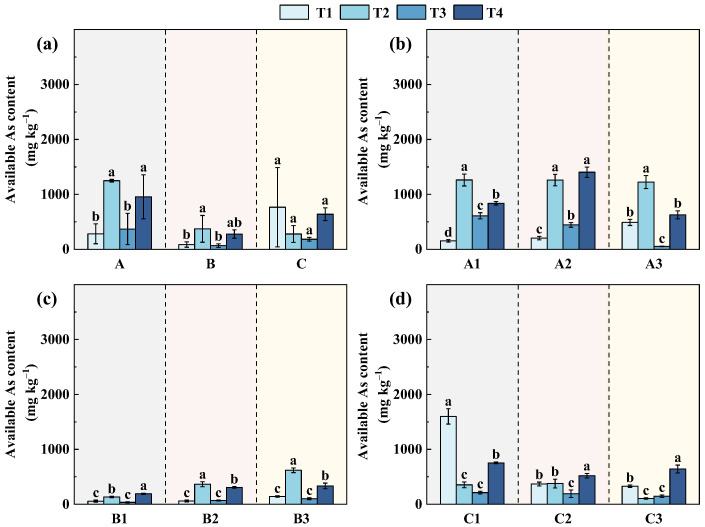
Variations in available As (A-As) concentrations at different sampling stages: (**a**) overall comparison among slopes A–C; (**b**) slope A; (**c**) slope B; and (**d**) slope C. T1, one year before remediation; T2, immediately after remediation; T3, six months after remediation; T4, one and a half years after remediation. A1–A3, B1–B3, and C1–C3 denote the three sampling points on slopes A, B, and C, respectively. Different lowercase letters indicate significant differences among sampling times at the same site (*p* < 0.05).

Total As reflects overall contamination, whereas the available fraction indicates its mobility and potential ecological relevance. As shown in Figure 3 and Figure S2, available As increased from T1 to T4 at all slopes, with the magnitude following the order slope A > slope B > slope C. At slope A, available As ranged from approximately 200–800 mg·kg^−1^, accounting for 1–4% of total As, despite the highest total As concentrations at this site, which is likely associated with its gentle topography (<15 m elevation difference) and limited lateral transport. These conditions favor local retention of As and promote its stabilization within the soil matrix, resulting in high total As but only a small fraction occurring in available forms [41]. Following remediation, all sampling points exhibited a similar temporal pattern, characterized by an initial increase and subsequent decline. Specifically, available As increased markedly from 153–489 mg·kg^−1^ before remediation to 1223–1262 mg·kg^−1^ at T2, corresponding to a 2.5–8.2-fold increase. This pronounced rise suggests that engineering activities, including soil excavation, backfilling, and surface reconstruction, substantially disturbed the original As-binding phases and promoted the transformation of relatively stable As into more labile forms. With continued remediation, available As declined to 444–610 mg·kg^−1^ at T3 and further decreased or stabilized at 628–1403 mg·kg^−1^ at T4, reflecting gradual recovery of soil structure and enhanced immobilization driven by amendment application and vegetation establishment. Nevertheless, available As remained higher than pre-remediation levels throughout the monitoring period, suggesting that As speciation had not yet fully re-equilibrated. This indicates that the stabilization process operates on a longer timescale and highlights the persistent influence of remediation-induced disturbance on As mobility, demonstrating the necessity of extended monitoring to accurately evaluate remediation effectiveness and environmental risk.

At slope B, available As exhibited a clear stage-dependent variation across the three sampling sites (B1–B3) (Figure 3c). At T1, available As levels were relatively low, ranging from 56.37 to 142.00 mg·kg^−1^. At T2, available As increased markedly at all sites, reaching 132.37, 364.83, and 619.27 mg·kg^−1^ at B1, B2, and B3, respectively, corresponding to a 2.3–4.4-fold increase. This increase indicates that engineering disturbance associated with soil excavation and slope reconstruction substantially enhanced As mobilization during the early remediation stage. Subsequently, available As concentrations declined to 35.12–100.51 mg·kg^−1^ at T3, approaching or even falling below pre-remediation levels, suggesting that the combined effects of surface soil removal, soil amendment application, and terraced slope stabilization effectively suppressed As mobility. However, available As exhibited a moderate rebound at T4, increasing to 191.15–334.88 mg·kg^−1^, although remaining substantially lower than that in T2. This illustrates that while engineering and revegetation measures effectively reduced short-term As activation, longer-term processes such as rainfall infiltration, slope runoff redistribution, and rhizosphere interactions may still promote partial reactivation of As [18,47,48,49].

At slope C, available As exhibited pronounced stage-dependent variations during the remediation process, showing a distinct pattern compared with Areas A and B (Figure 3d). Available As contents were relatively high at T1, reaching 1599.23, 369.65, and 328.97 mg·kg^−1^ at sites C1, C2, and C3, respectively, reflecting the strong mobilization of As under acidic conditions. After remediation, available As decreased markedly at all sites to 354.47, 377.53, and 104.67 mg·kg^−1^, representing reductions of approximately 65–85% relative to pre-remediation levels at T2. This sharp decline shows that surface soil removal, alkaline amendments (e.g., clam and red mud), and hydrological regulation effectively limited As mobility during the early remediation stage. After six months of remediation (T3), available As further decreased to 146.28–212.36 mg·kg^−1^, suggesting a temporary stabilization of As under improved soil chemical conditions. However, available As increased again to 753.96, 519.88, and 642.23 mg·kg^−1^ at C1–C3 after one year of remediation (T4), respectively. Although these values remained lower than those at T1, the rebound indicates that As remobilization occurred under long-term hydrological disturbance and sediment redistribution, highlighting the strong control of water–soil interactions on As availability and the inherent instability of remediation outcomes in hydrologically active environments.

### 3.2. Changes in Soil Physicochemical Properties

#### 3.2.1. Variation in Soil pH

**Figure 4:**
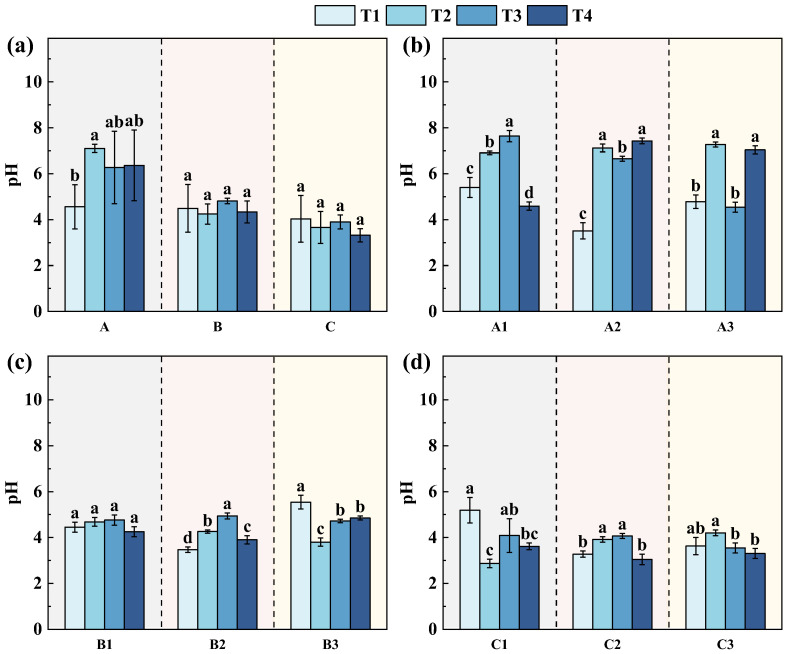
pH changes at different sampling stages: (**a**) overall comparison among slopes A–C; (**b**) slope A; (**c**) slope B; and (**d**) slope C. T1, one year before remediation; T2, immediately after remediation; T3, six months after remediation; T4, one and a half years after remediation. A1–A3, B1–B3, and C1–C3 denote the three sampling points on slopes A, B, and C, respectively. Different lowercase letters indicate significant differences among sampling times at the same site (*p* < 0.05).

During the remediation process, soil pH exhibited clear stage-dependent variations across the three slopes, with pronounced differences in both magnitude and stability among sites (Figure 4a–d). At T1, soils in slopes A, B, and C were uniformly acidic, with pH values ranging from 4.50 to 5.00 and no significant differences among the sites. After remediation, soil pH in slope A increased markedly, whereas only minor changes were observed in slopes B and C, and these differences were not statistically significant. This pattern indicates a spatially heterogeneous response of soil acid–base conditions to the applied remediation measures. Specifically, soil pH in slope A increased from 4.56 before remediation (T1) to 7.10 immediately after remediation (T2), reflecting rapid neutralization of acidic conditions. Subsequently, pH values remained near neutral during the post-remediation monitoring period (6.27–6.36 at T3–T4), indicating relatively good macroscopic stability. Nevertheless, notable spatial heterogeneity persisted within Area A: although all sampling points (A1–A3) reached near-neutral pH at T2 (approximately 6.91–7.27), some locations exhibited pronounced declines at later stages (e.g., A3 decreased to 4.54 at T3 and A1 to 4.59 at T4), suggesting localized influences such as residual acid sources, rainfall-induced leaching, or material redistribution.

The increase in soil pH may suppress the dissolution of Fe/Al oxide phases and promote As adsorption or co-precipitation, thereby contributing to reduced As mobility (Figure 4b). In this study, such effects are likely linked to the application of alkaline amendments (e.g., CaO), which rapidly neutralized soil acidity and may have facilitated the formation of relatively insoluble Ca–As phases (e.g., Ca_3_(AsO_4_)_2_, Ca_3_(AsO_3_)_2_ and CaHAsO_4_·H_2_O). Additionally, the incorporation of red mud, rich in Fe/Al oxides, likely provided supplementary adsorption sites for arsenic, enhancing its retention. Vegetation restoration may also have modulated As dynamics through organic matter inputs and alterations in soil structure, potentially influencing surface complexation processes [50,51,52].

As for slopes B and C (Figure 4c,d), soil pH also exhibited clear stage-dependent variations during the remediation process, although both the magnitude and stability of these changes were lower than those observed at slope A. At slope B, soil pH ranged from 3.47 to 5.54 prior to remediation and showed a slight increase after treatment, reaching its highest level at six months (4.76–4.94), followed by a minor decline after one year while remaining comparable to or slightly higher than that in T1. This pattern indicates a moderate but relatively stable pH adjustment under the combined effects of engineering treatment and subsequent soil equilibration. In contrast, slope C exhibited lower pH values and stronger temporal fluctuations. Although soil pH increased temporarily after remediation, reaching approximately 4.00–4.70 at the mid-term stage, it declined again after one year to 3.30–3.60, approaching pre-remediation levels. This suggests that the amelioration of soil acidity in slope C was short and strongly constrained by hydrological conditions and continuous acid input. The different changes in pH among three slopes are consistent with the observed spatial differences in available As behavior, highlighting soil pH as a key controlling factor governing As mobility and stability under different remediation settings.

#### 3.2.2. Variation in Soil Available Nitrogen (AN), Phosphorus (AP), and Potassium (AK)

**Figure 5:**
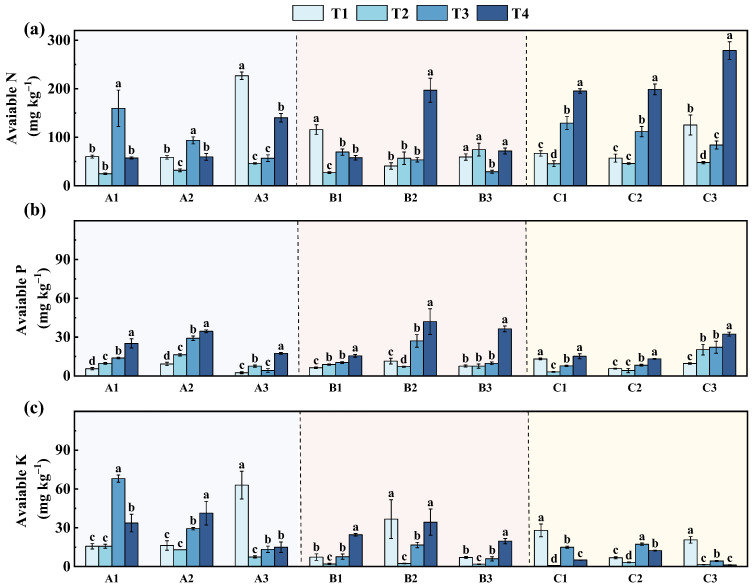
Variations in soil available nutrients at different sampling stages across slopes A1–C3: (**a**) AN; (**b**) AP; (**c**) AK. T1, one year before remediation; T2, immediately after remediation; T3, six months after remediation; T4, one and a half years after remediation. Different lowercase letters indicate significant differences among sampling times at the same site (*p* < 0.05).

Prior to remediation, the mean concentrations of AN (Figure 5a), AP, and AK in the mining area were 90.07 ± 57.15, 7.88 ± 3.36, and 24.74 ± 18.08 mg·kg^−1^, respectively, indicating generally poor soil fertility as a consequence of long-term mining disturbance. After remediation, soil nutrient levels generally increased across all slopes, reflecting an overall improvement in nutrient availability in the mining area. However, the extent of this improvement and the timing of nutrient responses varied considerably among different nutrients and across sites.

Among the three nutrients, AN showed the most obvious increase and the widest variation (Figure 5a). Across slopes A, B, and C, AN concentrations ranged from approximately 30 to 280 mg·kg^−1^. At slope A, AN levels mainly ranged from 25 to 230 mg·kg^−1^, with relatively higher values observed under the T3 and T1 treatments; notably, the AN content at site A3 under T1 exceeded 220 mg·kg^−1^. At slope B, AN contents were generally lower than those at slopes A and C, mostly distributed within 30–200 mg·kg^−1^, although a marked increase was observed under the T4 treatment, with the value at site B2 approaching 200 mg·kg^−1^. By contrast, area C exhibited the highest overall AN levels, ranging from 45 to 280 mg·kg^−1^, with the maximum value recorded at site C3 under T4. This behavior is mainly related to the strongly acidic conditions, which can suppress microbial activity and slow down nitrogen mineralization and transformation, thereby promoting the accumulation of nitrogen derived from plant and organic residues in the soil [53].

AP displayed a similar but less pronounced response trend (Figure 5b). After remediation, AP contents at several sites were relatively low, mostly below 10 mg·kg^−1^, which is generally considered below the optimal range for agricultural soils. Following remediation, AP contents increased substantially across all areas. At slope A, AP contents rose from approximately 2–10 mg·kg^−1^ to 15–35 mg·kg^−1^, with the highest values observed at site A2. At slope B, AP exhibited a wider variation (6–56 mg·kg^−1^), and a marked increase was observed at site B2 during the later treatment stage. Compared with slopes A and B, slope C showed relatively moderate AP levels, generally ranging from ~3 to 33 mg·kg^−1^, although an upward trend was also evident, particularly at site C3. These results suggest that AP was initially limited but was markedly improved following remediation, with several sites reaching relatively high AP levels (>40 mg·kg^−1^).

AK exhibited comparatively moderate variability across the study slopes (Figure 5c). In general, AK contents ranged from approximately 4 to 75 mg·kg^−1^. At slope A, AK values showed relatively large fluctuations (12–75 mg·kg^−1^), with the highest level observed at site A3. At slope B, AK contents were generally lower, mostly within 4–50 mg·kg^−1^, although a noticeable increase occurred at site B2 during the later treatment stage. Slope C showed relatively stable AK levels, primarily within 18–34 mg·kg^−1^, with smaller variations among treatments. From an agronomic perspective, AK levels at several sites prior to remediation were at low to moderate levels, which could constrain K availability for crop growth [54,55,56]. Following remediation, AK contents increased to ranges generally regarded as adequate for agricultural soils. It should be noted that variations in nutrient concentrations cannot be interpreted in isolation. Disturbance to soil microbial communities can induce short-term increases in nutrient availability, which may mask underlying instability. Accordingly, a comprehensive evaluation based on multiple soil indicators is required to reliably assess soil quality changes.

#### 3.2.3. Variation in Soil Organic Matter and Cation Exchange Capacity

**Figure 6:**
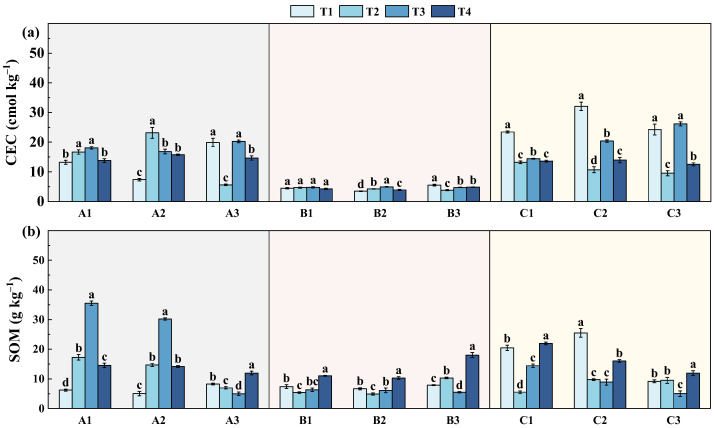
Variations in soil cation exchange capacity (CEC) and soil organic matter (SOM) at different sampling stages across slopes A1–C3: (**a**) cation exchange capacity (CEC); (**b**) soil organic matter (SOM); T1, one year before remediation; T2, immediately after remediation; T3, six months after remediation; T4, one and a half years after remediation. Different lowercase letters indicate significant differences among sampling times at the same site (*p* < 0.05).

To further assess the improvement in soil quality following remediation, cation exchange capacity (CEC) and soil organic matter (SOM), which are key indicators of soil nutrient retention and fertility, were subsequently examined (Figure 6). The CEC values in slopes A, B, and C exhibited distinct variations under different treatments, with overall values ranging from 5 to 30 cmol·kg^−1^. In slope A, CEC showed a clear increasing trend with remediation progress, and values at the later sampling stages were generally higher than those at the initial stage, indicating an improvement in CEC during remediation. In contrast, slope B exhibited relatively low CEC values, mainly distributed within 5–12 cmol·kg^−1^, and the variation before and after remediation was limited, with only a moderate increase observed at site B2. At slope C, CEC values were generally at a moderate level, ranging from 10–20 cmol·kg^−1^. Notably, CEC in slope C was relatively high at the early stage of remediation but showed a certain decline during the remediation process, which may be associated with the addition of passivating agents, which can regulate soil pH and promote the fixation of metal cations, thereby reducing the amount of exchangeable cations. With prolonged remediation, CEC exhibited a gradual increasing trend, possibly due to the aging of the passivating materials and the partial release of exchangeable cations.

As for SOM, the variation also showed distinct regional characteristics during remediation. At slope A, SOM increased markedly after remediation and reached its maximum at the mid-term stage, with values rising from approximately 5–8 g·kg^−1^ before remediation to 30–35 g·kg^−1^ at T3, followed by a moderate decline at T4. This trend suggests that organic inputs or improved soil conditions promoted SOM accumulation at the early stage, whereas enhanced microbial activity and organic matter mineralization may have contributed to the subsequent decrease. At slope B, SOM exhibited relatively small fluctuations throughout the remediation process. Initial SOM levels were low (approximately 6–8 g·kg^−1^), and only a moderate increase was observed at the late stage, with values rising to 10–18 g·kg^−1^ at T4. This limited response indicates that organic matter accumulation in this slope was less sensitive to remediation measures, possibly due to lower organic inputs or weaker vegetation recovery. In contrast, slope C showed a different SOM evolution behavior. SOM content was relatively high before remediation (19–26 g·kg^−1^), but decreased sharply during the early stage (T2: 5–10 g·kg^−1^). Subsequently, SOM gradually increased, reaching 11–22 g·kg^−1^ at T4, comparable to or slightly higher than pre-remediation levels. (Figure 6b) Given that slope C is located adjacent to an acidic hydrological environment, the lower pH conditions may suppress microbial activity and slow organic matter decomposition, thereby favoring SOM accumulation. The relatively high SOM may also contribute to enhanced retention of arsenic through sorption or complexation processes, potentially reducing its mobility.

#### 3.2.4. Correlation Analysis

**Figure 7:**
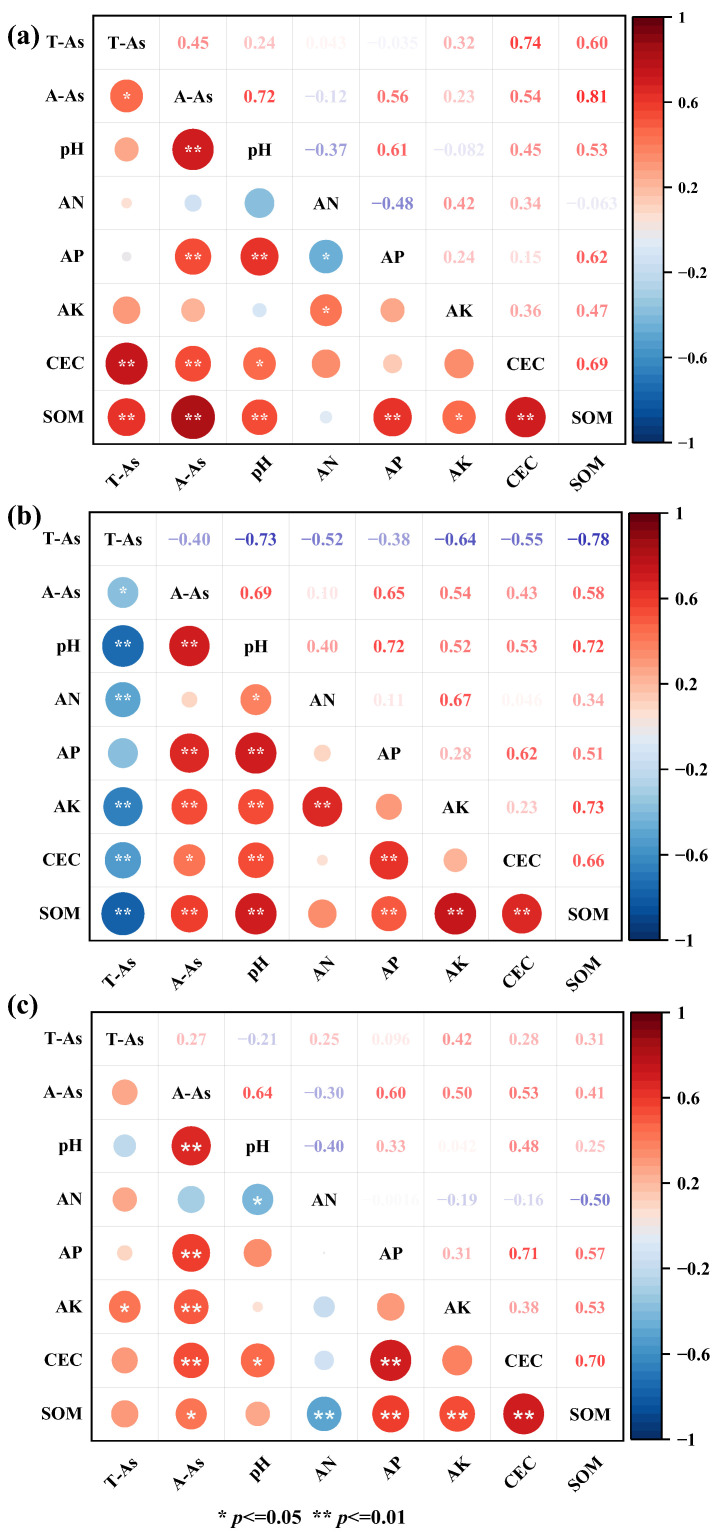
Pearson correlation heatmaps showing the relationships among changes in soil properties and As fractions at different remediation stages: (**a**) ΔT2–T1, representing changes immediately after remediation; (**b**) ΔT3–T1, representing changes six months after remediation; (**c**) ΔT4–T1, representing changes one and a half years after remediation. Asterisks denote significant correlations (*p* < 0.05; *p* < 0.01). T-As and A-As denote total As and available As.

Static correlation matrices (Figures S3–S6) indicate significant correlations between total As and pH, SOM, CEC, and nutrient (AN, AP, AK) changes across sampling stages. Correlation analyses based on remediation stages (ΔT2–T1, ΔT3–T1, and ΔT4–T1) were further performed to capture the response of As to soil property changes during remediation (Figure 7). At the early remediation stage (ΔT2–T1) (Figure 7a), changes in total As (T-As) showed strong positive correlations with available As (A-As), indicating that initial engineering disturbance primarily affected As redistribution rather than long-term stabilization. During this period, pH exhibited a significant negative correlation with A-As, suggesting that the rapid increase in soil pH after amendment application effectively reduced As mobility. However, correlations between As and nutrient-related parameters were relatively weak, implying that nutrient recovery had not yet become a dominant controlling factor. At six months after remediation (ΔT3–T1) (Figure 7b), correlations among variables became more extensive. The negative relationship between pH and A-As remained significant, while stronger positive correlations emerged between A-As and SOM as well as CEC. This pattern indicates that, with progressing remediation, As behavior was increasingly regulated by soil adsorption capacity rather than solely by pH-driven desorption. Meanwhile, AN and AK showed moderate correlations with As, reflecting the gradual coupling between nutrient dynamics and As stabilization.

At 1.5 years after remediation (ΔT4–T1) (Figure 7c), SOM and CEC were significantly significant negatively correlated with A-As, whereas their relationships with T-As weakened, indicating that soil colloidal adsorption and organic matter accumulation may play an increasingly important role in As immobilization. In contrast, the influence of pH weakened relative to earlier stages, whereas CEC, SOM, and nutrient indicators (AN, AP, and AK) showed stronger associations with As mobility, suggesting that As dynamics remained responsive to changes in soil conditions and highlighting the importance of continued monitoring.

These findings suggest that the effectiveness of mine remediation depends on long-term interactions between site geomorphological conditions and applied remediation strategies. In low-relief slopes dominated by slope regulation and revegetation, monitoring frequency should be increased, with assessments conducted at least every six months and maintained for more than 1.5 years, accompanied by adaptive remediation measures as needed. In contrast, in areas with greater topographic relief, engineering measures such as interception and filtration contribute to more stable remediation outcomes. However, in areas adjacent to acidic water bodies, remediation may show short-term effectiveness but lack long-term stability. Real-time monitoring of soil pH is therefore recommended, as a decline in pH may lead to renewed increases in available As and associated environmental risks.

## 4. Conclusions

This two-year field study demonstrates that remediation of As-contaminated mining soils leads to pronounced but non-linear changes in soil geochemistry and As mobility. Engineering-based interventions triggered short-term redistribution of arsenic, with total As temporarily increasing by up to 1.6–8.7 times in disturbed surface soils, followed by gradual stabilization within approximately one year. In contrast, areas influenced by acidic and hydrologically active conditions exhibited substantial early reductions in total As (>70%) and available As (65–85%), yet later rebounds highlight the temporal instability of remediation effects. Across the site, variations in pH, SOM, and CEC were closely associated with changes in available As, confirming the coupled geochemical and hydrological regulation of arsenic behavior. From a management perspective, remediation in relatively stable geomorphic settings should consider local rainfall regimes to minimize potential off-site transport following disturbance, whereas areas affected by acidic water inputs would benefit from enhanced monitoring of soil and water pH to enable early detection of conditions that may increase metal mobility. These findings underscore the dynamic and site-specific nature of arsenic stabilization and highlight the importance of long-term monitoring for adaptive remediation and environmental risk management. However, the present study provides primarily semi-quantitative interpretations based on field observations, and future research integrating process-based monitoring, mineralogical analyses and microbial investigations is needed to further elucidate the mechanisms governing arsenic transformation.

## Figures and Tables

**Table 1:** Characteristics of different slope areas before remediation and corresponding remediation measures.

Site	Pre-Remediation Characteristics	Remediation Measures
Slope A	Small elevation difference (<15 m), surface covered with bluish-black contaminated residues (~10 cm), and partial vegetation cover.	Slope reshaping + soil amendment + vegetation restoration (landscape vegetation), drainage system improvement + ecological ditches, and runoff control (sedimentation ponds and check dams).
Slope B	Large elevation difference (~52.7 m), surface residues of bluish-black tailings (~10 cm), and partial vegetation cover.	Surface-contaminated soil removal + terraced slope construction + soil amendment + vegetation restoration (grass-dominated communities).
Slope C	Located near a water body; strongly acidic conditions (pH ≈ 3.0); small elevation difference; surface accumulation of tailings and sludge; tailings deposited in adjacent ponds; sparse vegetation cover.	Tailings relocation and containment + stepped filtration system + slope vegetation restoration (grass-dominated communities with landscape vegetation).

## Data Availability

The original contributions presented in this study are included in the article/Supplementary Material. Further inquiries can be directed to the corresponding authors.

## Supplementary Materials

The following supporting information can be downloaded at https://www.mdpi.com/article/10.3390/toxics14040316/s1. Figure S1: Total As content at T1 and T4 at slope A (a), slope B (b), and slope C (c); Figure S2: Available As content at T1 and T4 at slope A (a), slope B (b), and slope C (c); Figure S3: Pearson correlation heatmaps showing the relationships among changes in soil properties and arsenic fractions at T1; Figure S4: Pearson correlation heatmaps showing the relationships among changes in soil properties and arsenic fractions at T2; Figure S5: Pearson correlation heatmaps showing the relationships among changes in soil properties and arsenic fractions at T3; Figure S6: Pearson correlation heatmaps showing the relationships among changes in soil properties and arsenic fractions at T4; Table S1: Pre-remediation characteristics and remediation measures for different slope areas.

## References

[1] Sonter L.J., Herrera D., Barrett D.J., Galford G.L., Moran C.J., Soares B.S. (2017). Mining drives extensive deforestation in the Brazilian Amazon. Nat. Commun..

[2] Jones D.O.B., Arias M.B., Van Audenhaege L., Blackbird S., Boolukos C., Bribiesca-Contreras G., Copley J.T., Dale A., Evans S., Fleming B.F.M. (2025). Long-term impact and biological recovery in a deep-sea mining track. Nature.

[3] Beylot A., Bodenan F., Guezennec A.G., Muller S. (2022). LCA as a support to more sustainable tailings management: Critical review, lessons learnt and potential way forward. Resour. Conserv. Recycl..

[4] Rana N.M., Ghahramani N., Evans S.G., Small A., Skermer N., McDougall S., Take W.A. (2022). Global magnitude-frequency statistics of the failures and impacts of large water-retention dams and mine tailings impoundments. Earth-Sci. Rev..

[5] das Neves M.D.B., Gama M.A.P., Ishihara J.H., da Silva D.P., Ferreira G.C., Noronha N.C., Sanchez L.E., Paschoal J.P. (2024). Closure process of bauxite tailings facilities: The induction of ecological succession can enhance substrate quality in the initial phase of revegetation. Ecol. Eng..

[6] Leon R., Millán-Becerro R., Macías F., Cánovas C.R., Neculita C.M., Ayora C., Nieto J.M. (2026). Dispersed alkaline substrate passive treatment technology for highly contaminated acid mine drainage: 20 years of successful application. Water Res..

[7] Li Z.Y., Wang J., She Z.X., Gu J.Y., Lu H.Y., Wang S., He X., Yue Z.B. (2024). Tailings particle size effects on pollution and ecological remediation: A case study of an iron tailings reservoir. J. Hazard. Mater..

[8] Li Y.B., Guo L.F., Häggblom M.M., Yang R., Li M.Y., Sun X.X., Chen Z., Li F.B., Su X.F., Yan G. (2022). Are Responsible for Nitrogen Fixation Fueled by As(III) Oxidation, a Novel Biogeochemical Process Identified in Mine Tailings. Environ. Sci. Technol..

[9] Romero-Matos J., Cánovas C.R., Macías F., Pérez-López R., León R., Millán-Becerro R., Nieto J.M. (2023). Wildfire effects on the hydrogeochemistry of a river severely polluted by acid mine drainage. Water Res..

[10] Akhavan A., Golchin A. (2021). Estimation of arsenic leaching from Zn-Pb mine tailings under environmental conditions. J. Clean. Prod..

[11] Sanchez A.A., Haas K., Jackisch C., Hedrich S., Lau M.P. (2024). Enrichment of dissolved metal(loid)s and microbial organic matter during transit of a historic mine drainage system. Water Res..

[12] Xian L., Lu D., Zheng F., Fang J., Yang Y., Feng J., Wu D., Peñuelas J., Zeng S. (2026). Heavy rainfall shunts heavy metal transport from runoff to dominant sediment pathways in sludge-amended forest soils. J. Hazard. Mater..

[13] Lin C.Y., Ali B.N.M., Tair R., Musta B., Abdullah M.H., Cleophas F., Isidore F., Nadzir M.S.M., Roselee M.H., Yusoff I. (2022). Distance impacts toxic metals pollution in mining affected river sediments. Environ. Res..

[14] Mensah A.K., Marschner B., Wang J.X., Bundschuh J., Wang S.L., Yang P.T., Shaheen S.M., Rinklebe J. (2022). Reducing conditions increased the mobilisation and hazardous effects of arsenic in a highly contaminated gold mine spoil. J. Hazard. Mater..

[15] Stolze L., Battistel M., Rolle M. (2022). Oxidative Dissolution of Arsenic-Bearing Sulfide Minerals in Groundwater: Impact of Hydrochemical and Hydrodynamic Conditions on Arsenic Release and Surface Evolution. Environ. Sci. Technol..

[16] Alvarez-Ayuso E. (2022). Stabilization and encapsulation of arsenic-/antimony-bearing mine waste: Overview and outlook of existing techniques. Crit. Rev. Environ. Sci. Technol..

[17] Tanaskovski B., Petrović M., Kljajić Z., Degetto S., Stanković S. (2014). Analysis of major, minor and trace elements in surface sediments by X-ray fluorescence spectrometry for assessment of possible contamination of Boka Kotorska Bay, Montenegro. Maced. J. Chem. Chem. Eng..

[18] Smedley P.L., Kinniburgh D.G. (2002). A review of the source, behaviour and distribution of arsenic in natural waters. Appl. Geochem..

[19] Nordstrom D.K. (2002). Worldwide occurrences of arsenic in ground water. Science.

[20] Proto M., Courtney R. (2023). Application of organic wastes to subsoil materials can provide sustained soil quality in engineered soil covers for mine tailings rehabilitation: A 7 years study. Ecol. Eng..

[21] Xu H.L., Qiao X., Gao G.K., Dou H.T., Waheed A., Aili A. (2026). Advances in ecological restoration of mining-impacted landscapes: Techniques, case studies, and key challenges. Environ. Res..

[22] Wen X.C., Zhou J.W., Zheng S.Y., Yang Z.W., Lu Z., Jiang X.Q., Zhao L.Z., Yan B., Yang X.F., Chen T. (2024). Geochemical properties, heavy metals and soil microbial community during revegetation process in a production Pb-Zn tailings. J. Hazard. Mater..

[23] Tibbett M. (2024). Post-mining ecosystem reconstruction. Curr. Biol..

[24] Zhu F.Y., Zhang W.C., Puzrin A.M. (2024). The slip surface mechanism of delayed failure of the Brumadinho tailings dam in 2019. Commun. Earth Environ..

[25] Rotta L.H.S., Alcântara E., Park E., Negri R.G., Lin Y.N., Bernardo N., Mendes T.S.G., Souza C.R. (2020). The 2019 Brumadinho tailings dam collapse: Possible cause and impacts of the worst human and environmental disaster in Brazil. Int. J. Appl. Earth Obs..

[26] Morin G., Juillot F., Casiot C., Bruneel O., Personné J.-C., Elbaz-Poulichet F., Leblanc M., Ildefonse P., Calas G. (2003). Bacterial Formation of Tooeleite and Mixed Arsenic(III) or Arsenic(V)−Iron(III) Gels in the Carnoulès Acid Mine Drainage, France. A XANES, XRD, and SEM Study. Environ. Sci. Technol..

[27] Casiot C., Morin G., Juillot F., Bruneel O., Personné J.-C., Leblanc M., Duquesne K., Bonnefoy V., Elbaz-Poulichet F. (2003). Bacterial immobilization and oxidation of arsenic in acid mine drainage (Carnoulès creek, France). Water Res..

[28] Xie W.J., Che L., Zhou G.Y., Yang L.N., Hu M.Y. (2010). The bioconcentration ability of heavy metals in 50 rice cultivars under identical experimental conditions. Environ. Sci..

[29] (2007). Determination of pH in Soil.

[30] (2006). Soil Testing—Part 6: Method for Determination of Soil Organic Matter.

[31] (1999). Determination of Cation Exchange Capacity in Forest Soils.

[32] (2015). Nitrogen Determination Methods of Forest Soils.

[33] (2014). Soil Testing—Part 7: Method for Determination of Available Phosphorus in Soil.

[34] (2004). Soil Testing—Method for Determination of Available and Slowly Available Potassium in Soil.

[35] (2008). Soil Quality-Analysis of Total Mercury, Arsenic and Lead Contents in Soils-Atomic Fluorescence Spectrometry-Part 2: Analysis of Total Arsenic Contents in Soils.

[36] Kim J.-Y., Davis A.P., Kim K.-W. (2003). Stabilization of Available Arsenic in Highly Contaminated Mine Tailings Using Iron. Environ. Sci. Technol..

[37] Yang Y.P., Wang P., Yan H.J., Zhang H.M., Cheng W.D., Duan G.L., Zhu Y.G. (2019). NH_4_H_2_PO_4_-extractable arsenic provides a reliable predictor for arsenic accumulation and speciation in pepper fruits (*Capsicum annum* L.). Environ. Pollut..

[38] Palmer M.J., Richardson M., Chételat J., Spence C., Connon R., Jamieson H.E. (2024). Watershed hydrology mediates the recovery of an arsenic impacted subarctic landscape. Environ. Pollut..

[39] Park J., Lee D.Y., Kim H., Woo N.C. (2023). Effects of dry and heavy rainfall periods on arsenic species and behaviour in the aquatic environment adjacent a mining area in South Korea. J. Hazard. Mater..

[40] Hancock G.R., Duque J.F.M., Willgoose G.R. (2020). Mining rehabilitation—Using geomorphology to engineer ecologically sustainable landscapes for highly disturbed lands. Ecol. Eng..

[41] Madejón P., Caro-Moreno D., Navarro-Fernández C.M., Rossini-Oliva S., Marañón T. (2021). Rehabilitation of waste rock piles: Impact of acid drainage on potential toxicity by trace elements in plants and soil. J. Environ. Manage..

[42] Zhang H., Selim H.M. (2005). Kinetics of arsenate adsorption-desorption in soils. Environ. Sci. Technol..

[43] Feng Q., Zhang Z., Chen Y., Liu L., Zhang Z., Chen C. (2013). Adsorption and desorption characteristics of arsenic on soils: Kinetics and equilibrium. Procedia Environ. Sci..

[44] Lu S., Wang Y., Zhang J., Liu X., Chen H., Li W. (2022). Arsenic adsorption and desorption in various aqueous media. Sustainability.

[45] Jiang Q., He Y.M., Wu Y.L., Dian B., Zhang J.L., Li T.G., Jiang M. (2022). Solidification/stabilization of soil heavy metals by alkaline industrial wastes: A critical review. Environ. Pollut..

[46] Hong Y.K., Kim J.W., Lee S.P., Yang J.E., Kim S.C. (2022). Effect of Combined Soil Amendment on Immobilization of Bioavailable As and Pb in Paddy Soil. Toxics.

[47] Hammond C.M., Root R.A., Maier R.M., Chorover J. (2020). Arsenic and iron speciation and mobilization during phytostabilization of pyritic mine tailings. Geochim. Cosmochim. Acta.

[48] Zarzsevszkij S., Vítková M., Pospísková K.Z., Kolarík J., Hudcová B.B., Jurkovic L. (2024). Management of a contaminated mine soil: Effect of soil water content on antimony and arsenic immobilisation by iron-based amendments and biochar composites. Soil Use Manag..

[49] Hammond C.M., Root R.A., Maier R.M., Chorover J. (2018). Mechanisms of Arsenic Sequestration by during the Phytostabilization of Metalliferous Mine Tailings. Environ. Sci. Technol..

[50] Rojas-Solis D., Larsen J., Lindig-Cisneros R. (2023). Arsenic and mercury tolerant rhizobacteria that can improve phytoremediation of heavy metal contaminated soils. PeerJ.

[51] Wenzel W.W., Kirchbaumer N., Prohaska T., Stingeder G., Lombi E., Adriano D.C. (2001). Arsenic fractionation in soils using an improved sequential extraction procedure. Anal. Chim. Acta.

[52] Dixit S., Hering J.G. (2003). Comparison of arsenic(V) and arsenic(III) sorption onto iron oxide minerals: Implications for arsenic mobility. Environ. Sci. Technol..

[53] Rousk J., Brookes P.C., Bååth E. (2009). Contrasting Soil pH Effects on Fungal and Bacterial Growth Suggest Functional Redundancy in Carbon Mineralization. Appl. Environ. Microb..

[54] Zörb C., Senbayram M., Peiter E. (2014). Potassium in agriculture—Status and perspectives. J. Plant Physiol..

[55] Römheld V., Kirkby E.A. (2010). Research on potassium in agriculture: Needs and prospects. Plant Soil.

[56] Wang M., Zheng Q.S., Shen Q.R., Guo S.W. (2013). The Critical Role of Potassium in Plant Stress Response. Int. J. Mol. Sci..

