# Supplementary material for: Responses of Arsenic and Soil Properties to Remediation: Evidence from a Two-Year Monitoring Study in an Abandoned Gold Mining Area
Source: Toxics. 2026 Apr 8;14(4):316. doi: 10.3390/toxics14040316 (PMC13120375; doi:10.3390/toxics14040316)
Supplement: Supplementary file 1 [file toxics-14-00316-s001.zip › toxics-4159355-supplementary.pdf]

Supporting Information

Responses of Arsenic and Soil Properties to Remediation: Evidence from a Two-Year Monitoring Study in an Abandoned Gold Mining Area

Zengling Tang[a], Lingyun Li[a], Yingyuting Li[a], Huayi Chen[c], Yili Zhang[a], Tian Hu[a, b*], Zheng Hu [a*]

[a] *Guangdong Research Center for Agricultural Soil Pollution Prevention and Control Engineering Technology, College of Natural Resources and Environment, South China Agricultural University, Guangzhou 510642, China*

[b] *School of Environmental Science and Engineering, Hainan University, Haikou 570228, Hainan Province, China*

[c] *School of Tropical Agriculture and Forestry, Hainan University, Haikou 570228, Hainan Province, China*

1. Determination of soil pH

Soil pH was determined in a 1:2.5 (w/v) soil-to-water suspension. Briefly, 4.0 g of air-dried soil (<2 mm) was mixed with 10 mL of boiled and cooled deionized water, shaken thoroughly, allowed to equilibrate for 30 min, and then measured using a calibrated pH meter.

2 Determination of available nitrogen

Soil available nitrogen (AN) was determined using the alkali diffusion method. Briefly, 2.00 g of air-dried soil passed through a 1 mm sieve was evenly spread in the outer chamber of a diffusion dish. Two milliliters of 2% boric acid solution and one drop of mixed indicator were added to the inner chamber. The edge of the outer chamber was then sealed with adhesive, and the diffusion dish was covered with a ground-glass lid. After slightly opening the lid to create a narrow gap, 10 mL of 1.0 $mol \cdot L^{-1}$ NaOH solution was quickly added to the outer chamber, and the dish was immediately sealed. The diffusion dish was then incubated at 40 °C for 24 h to allow the released ammonia to be absorbed by the boric acid solution. After incubation, the absorbed ammonia in the inner chamber was titrated with 0.005 $mol \cdot L^{-1}$ H_2SO_4 standard solution until the color changed from blue to red. The volume of sulfuric acid consumed was recorded. The content of alkali-hydrolyzable nitrogen in air-dried soil was calculated according to the following equation:

$$AN = C * V * 0.028 / m$$

where C is the concentration of the H_2SO_4 standard solution ($mol \cdot L^{-1}$), V is the volume of acid consumed during titration (mL), m is the mass of air-dried soil (g), and 0.028 represents the mass of nitrogen (g) contained in 1 mmol of NH_4^+.

3. Determination of available phosphorus

Soil available phosphorus was determined using sodium bicarbonate extraction

followed by the molybdenum–antimony colorimetric method (Olsen method). Briefly, 2.50 g of air-dried soil passed through a 20-mesh sieve was placed into a 150 mL Erlenmeyer flask. Fifty milliliters of 0.5 mol·L^{-1} $NaHCO_3$ solution and a small amount of phosphorus-free activated carbon were added to minimize interference from organic matter. The suspension was shaken at 20–25 °C for 30 min and immediately filtered, and the filtrate was collected for analysis. An aliquot of the filtrate (10 mL, or 2.5–5.0 mL for samples with high phosphorus concentrations, adjusted to 10 mL with 0.5 mol·L^{-1} $NaHCO_3$) was transferred into a 150 mL Erlenmeyer flask, followed by the addition of 35 mL deionized water and 5 mL of molybdenum–antimony reagent. After thorough mixing, the solution was allowed to develop color for 30 min. Absorbance was measured at 880 nm (or 700 nm, depending on instrument settings) using a reagent blank as the reference. A standard calibration curve was prepared using phosphorus standard solutions in the range of 0 – 0.5 μg·mL^{-1}. Soil available phosphorus concentrations were calculated based on the calibration curve and expressed on a dry-soil basis using the following equation.

$$P=\frac{\rho\times V\times t_s}{m\times 10^3\times k}\times 1000$$

where ρ is the phosphorus concentration determined from the standard curve (μg·mL^{-1}), V is the total volume of the color-developed solution (mL), t_s is the dilution factor (ratio of total extract volume to aliquot volume), m is the mass of air-dried soil (g), k is the conversion factor from air-dried to oven-dried soil, and 10^3 converts micrograms to milligrams.

4. Determination of available potassium

Soil available potassium was determined using ammonium acetate extraction followed by flame photometry. A series of potassium standard solutions was prepared, and the highest concentration standard was used to calibrate the flame photometer to

full scale (100). The standards were then measured sequentially from low to high concentration, and a calibration curve was established using potassium concentration ($\mu g \cdot mL^{-1}$) as the x-axis and instrument readings as the y-axis.

For sample analysis, 5.00 g of air-dried soil passed through a 1 mm sieve was placed in a 100 mL Erlenmeyer flask and extracted with 50 mL of 1.0 $mol \cdot L^{-1}$ NH_4OAc solution. The mixture was shaken for 30 min and subsequently filtered. The potassium concentration in the filtrate was determined using a flame photometer under the same conditions as the standards, and soil available potassium was calculated based on the calibration curve.

5. Determination of soil cation exchange capacity (CEC)

CEC was determined using the ammonium acetate exchange–distillation method. Briefly, 2.0 g of air-dried soil passed through a 2 mm sieve (5.0 g for light-textured soils) was placed into a 100 mL centrifuge tube and saturated with 1.0 $mol \cdot L^{-1}$ ammonium acetate (NH_4OAc) solution. The suspension was shaken thoroughly and centrifuged at 3000 ~ 4000 $r \cdot min^{-1}$ for 3 – 5 min, after which the supernatant was discarded. This extraction step was repeated 3 – 5 times until no Ca^{2+} was detected in the extract. The soil residue was subsequently washed with 95% ethanol several times to remove excess NH_4OAc until no NH_4^+ was detected in the wash solution (tested using Nessler's reagent). The washed soil was then transferred to a Kjeldahl flask, to which 50 – 80 mL of deionized water, 2 mL of liquid paraffin, and 1 g of MgO were added. The mixture was subjected to steam distillation for approximately 20 min. Ammonia released during distillation was absorbed in 25 mL of 20 $g \cdot L^{-1}$ boric acid solution containing a mixed indicator. The distillate was then titrated with standard hydrochloric acid solution. A blank determination was conducted in parallel, and the CEC was calculated based on the volume of acid consumed.

6. Determination of SOM

Soil organic matter was determined using the potassium dichromate oxidation method. Briefly, 0.5000 g of air-dried soil was weighed into a 500 mL Erlenmeyer flask. Subsequently, 10 mL of 1 $mol \cdot L^{-1}$ $K_2Cr_2O_7$ solution and 20 mL of concentrated H_2SO_4 were added sequentially. The flask was gently swirled for approximately 1 min to ensure thorough mixing and then allowed to stand on an asbestos board for 30 min to complete the oxidation reaction. After cooling, the reaction mixture was diluted to 250 mL with deionized water. Then, 12 – 15 drops of diphenylamine carboxylic acid indicator were added, and the excess dichromate was titrated with 0.5 $mol \cdot L^{-1}$ $FeSO_4$ standard solution until the solution color changed to grayish green. Alternatively, 3 – 4 drops of o-phenanthroline indicator were added, and titration was continued until the color changed from green to brick red, which was taken as the endpoint.

A blank determination was conducted following the same procedure without soil addition. If more than 75% of the added $K_2Cr_2O_7$ was reduced during titration, the soil mass was reduced and the analysis was repeated. Soil organic matter content was calculated based on the difference between the blank and sample titration volumes.

Table S1. Pre-remediation characteristics and remediation measures for different slope areas.

Site	Pre-remediation characteristics	Remediation measures
**Slope A**	Small elevation difference (< 15 m);<br>Surface covered by bluish-black contaminated residues (~10 cm);<br>Partial vegetation cover	**Surface contamination removal:** Removal of surface-contaminated residues and transfer to designated areas for stabilization and containment.<br>**Soil amendment:** Application of soil conditioners after slope reshaping (lime + red mud + iron salts) and addition of organic amendments (sludge/manure compost).<br>**Slope reshaping:** Gentle slope with east–west orientation; reshaped to a linear slope.<br>**Vegetation restoration:** Combination of shrubs and grasses (e.g., *Pennisetum, Bidens, salt-tolerant grasses, shrubs*).
**Slope B**	Large elevation difference (~52.7 m);<br>Surface covered by bluish-black mining residues (~10 cm);<br>Partial vegetation cover	**Surface contamination removal:** Same as Slope A.<br>**Soil amendment:** Same as Slope A.<br>**Slope reshaping:** Terraced slope with platform width of ~3 m and vertical spacing of ~6 m; step surfaces slightly inclined inward.<br>**Vegetation restoration:** Shrub–grass combination (e.g., *Pennisetum, Bidens, Medicago sativa, Leucaena leucocephala, Pinus massoniana*).
**Slope C**	Located near a pond;<br>Strongly acidic soil (pH ≈ 3.0);<br>Low elevation difference;<br>Accumulation of mining residues and sediments; Sparse vegetation cover	**Surface contamination removal:** Same as Slope A.<br>**Soil amendment:** Same as Slope A.<br>**Slope reshaping:** Gentle slope due to small elevation difference.<br>**Vegetation restoration:** *Grass-dominated restoration combined with landscape vegetation (e.g., Bidens, Pennisetum, Zinnia*).

Integrated remediation strategies:

Slope A: Slope reshaping + soil amendment + vegetation restoration (landscape-oriented combination) + drainage improvement + erosion control (sedimentation ponds and interception ditches). **Slope B:** Surface contamination removal + terraced slope construction + soil amendment + vegetation restoration (grass–shrub combination). **Slope C:** Transfer and containment of mine residues + stepwise filtration + slope stabilization + vegetation restoration (grass cover combined with landscape plants).

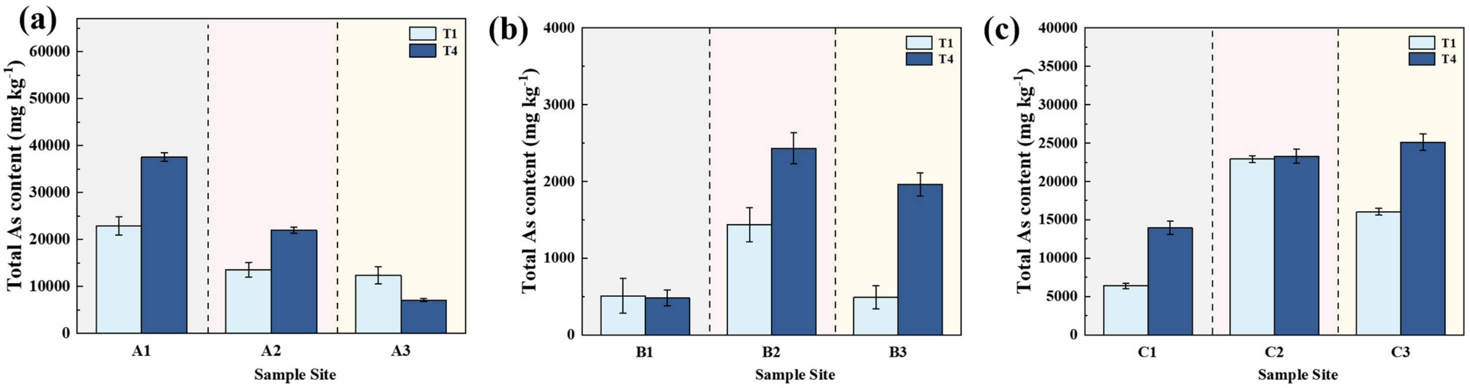

Figure S1. Total As content at T1 and T4 at slope A (a), slope B (b), and slope C (c),

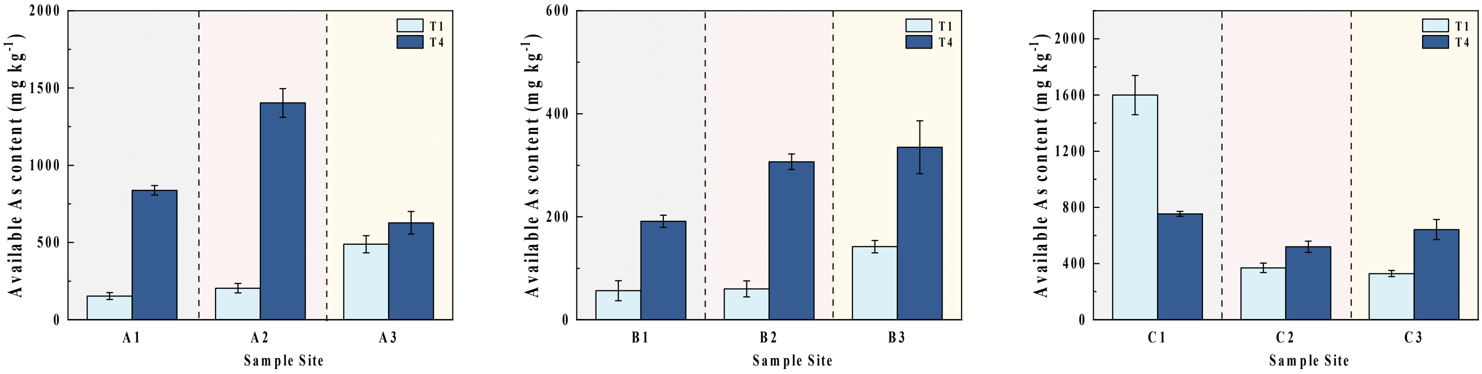

Figure S2. Available As content at T1 and T4 at slope A (a), slope B (b), and slope C (c).

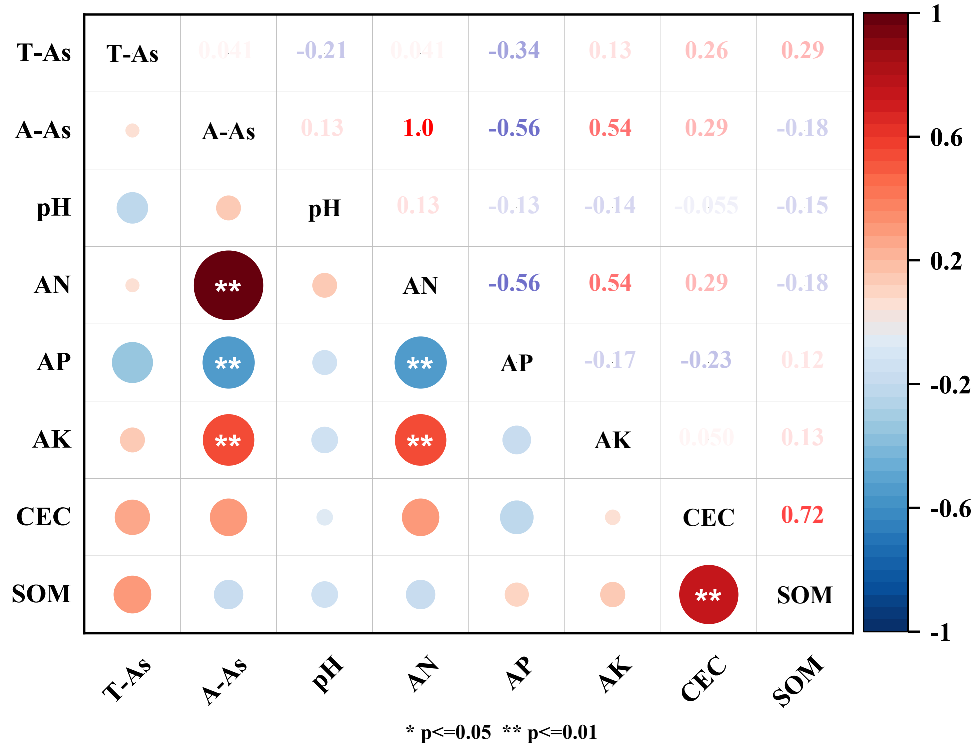

Figure S3. Pearson correlation heatmaps showing the relationships among changes in soil properties and arsenic fractions at T1.

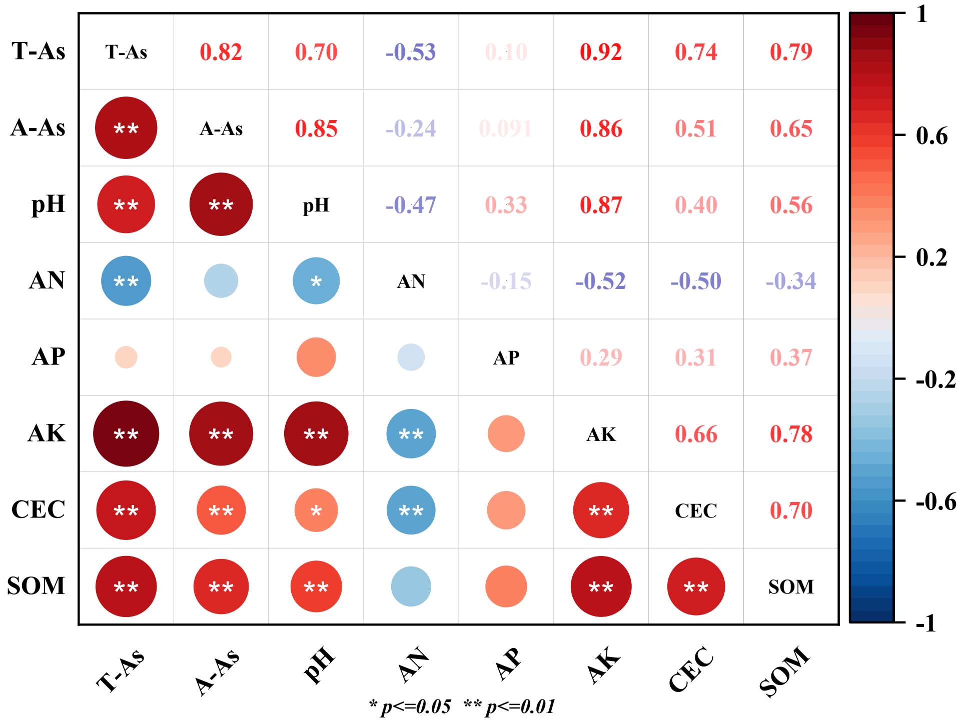

Figure S4. Pearson correlation heatmaps showing the relationships among changes in soil properties and arsenic fractions at T2.

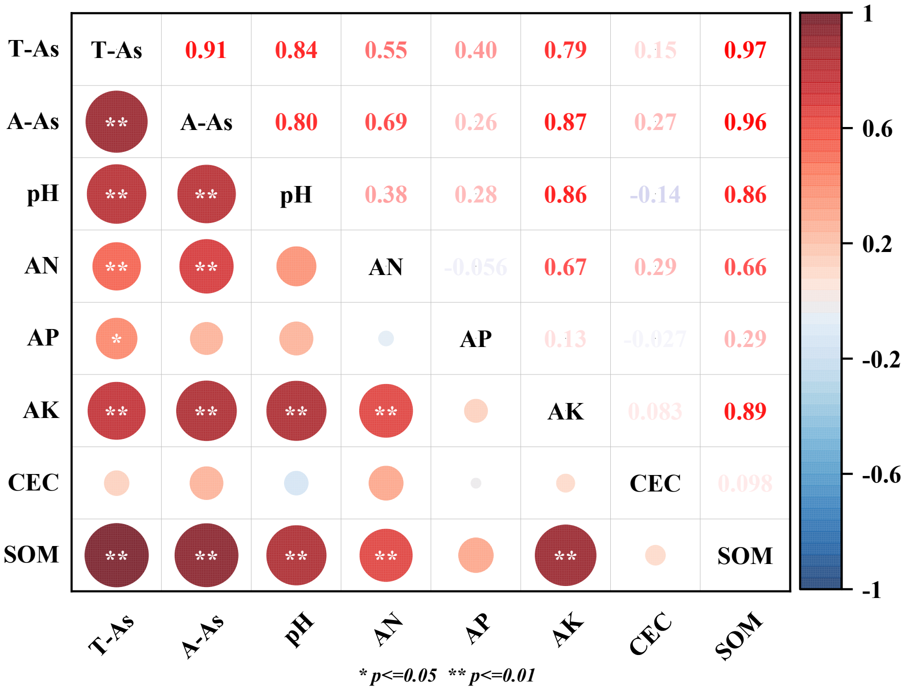

Figure S5. Pearson correlation heatmaps showing the relationships among changes in soil properties and arsenic fractions at T3.

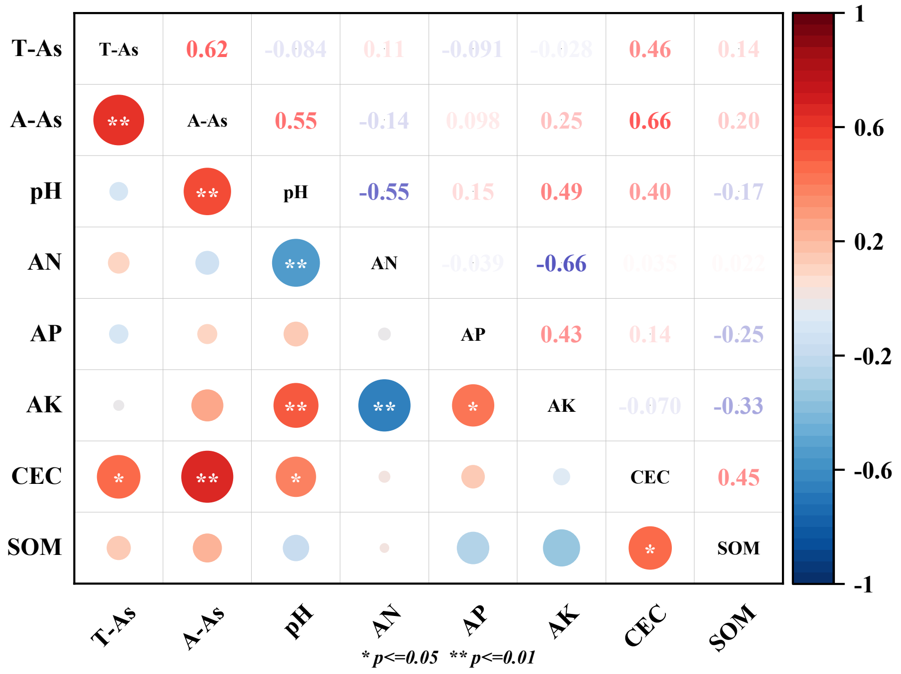

Figure S6. Pearson correlation heatmaps showing the relationships among changes in soil properties and arsenic fractions at T4.
